# Life-Threatening Docetaxel Toxicity in a Patient With Reduced-Function CYP3A Variants: A Case Report

**DOI:** 10.3389/fonc.2021.809527

**Published:** 2022-01-31

**Authors:** Nicholas R. Powell, Tyler Shugg, Reynold C. Ly, Costantine Albany, Milan Radovich, Bryan P. Schneider, Todd C. Skaar

**Affiliations:** ^1^ Department of Medicine, Division of Clinical Pharmacology, Indiana University School of Medicine, Indianapolis, IN, United States; ^2^ Department of Medicine, Division of Hematology/Oncology, Indiana University School of Medicine, Indianapolis, IN, United States

**Keywords:** DOCETAXEL, case report, CYP3A4, pneumonitis, pharmacogenetics

## Abstract

Docetaxel therapy occasionally causes severe and life-threatening toxicities. Some docetaxel toxicities are related to exposure, and inter-individual variability in exposure has been described based on genetic variation and drug-drug interactions that impact docetaxel clearance. Cytochrome P450 3A4 (CYP3A4) and CYP3A5 metabolize docetaxel into inactive metabolites, and this is the primary mode of docetaxel clearance. Supporting their role in these toxicities, increased docetaxel toxicities have been found in patients with reduced- or loss-of-function variants in *CYP3A4* and *CYP3A5*. However, since these variants in *CYP3A4* are rare, little is known about the safety of docetaxel in patients who are homozygous for the reduced-function *CYP3A4* variants. Here we present a case of life-threatening (grade 4) pneumonitis, dyspnea, and neutropenia resulting from a single dose of docetaxel. This patient was (1) homozygous for CYP3A4*22, which causes reduced expression and is associated with increased docetaxel-related adverse events, (2) heterozygous for CYP3A4*3, a rare reduced-function missense variant, and (3) homozygous for CYP3A5*3, a common loss of function splicing defect that has been associated with increased docetaxel exposure and adverse events. The patient also carried functional variants in other genes involved in docetaxel pharmacokinetics that may have increased his risk of toxicity. We identified one additional CYP3A4*22 homozygote that received docetaxel in our research cohort, and present this case of severe hematological toxicity. Furthermore, the one other CYP3A4*22 homozygous patient we identified from the literature died from docetaxel toxicity. This case report provides further evidence for the need to better understand the impact of germline *CYP3A* variants in severe docetaxel toxicity and supports using caution when treating patients with docetaxel who have genetic variants resulting in *CYP3A* poor metabolizer phenotypes.

## Introduction

Docetaxel is an antimicrotubular taxane derivative used for treatment of a variety of solid tumors. Due to its cytotoxicity, docetaxel occasionally causes severe and life-threatening toxicities (1). Both the incidence and severity of docetaxel-induced adverse events are related to exposure (2), and significant inter-individual variability in exposure has been described based on genetic variation and drug-drug interactions that impact docetaxel clearance (1, 3).

The drug-metabolizing enzymes and drug transporters that contribute to docetaxel pharmacokinetics are presented in **Supplemental Figure S1** (4). It is transported into hepatocytes *via* solute carrier organic anion transporter 1B3 (*SLCO1B3*) (5, 6) and excreted into the bile by multiple ATP-binding cassette (ABC) transporters (7). Within hepatocytes, cytochrome P450 3A4 (CYP3A4) and CYP3A5 metabolize docetaxel into inactive metabolites, and this is the primary mode of docetaxel clearance (1, 8). To avoid toxicities, the drug label recommends 50% reduction in dose when co-administered with strong CYP3A inhibitors (1). Similarly, pharmacogenetic evaluations have demonstrated increased docetaxel toxicities in patients with reduced- or loss-of-function variants in *CYP3A4* and *CYP3A5* (9, 10).

Pharmacogenetic guidelines for docetaxel have not been published yet, in part, due to the need for additional evidence. Here we present two cases that provide further evidence for a role of germline *CYP3A* genetics in severe docetaxel toxicity.

## Case Report

**Figure 1:**
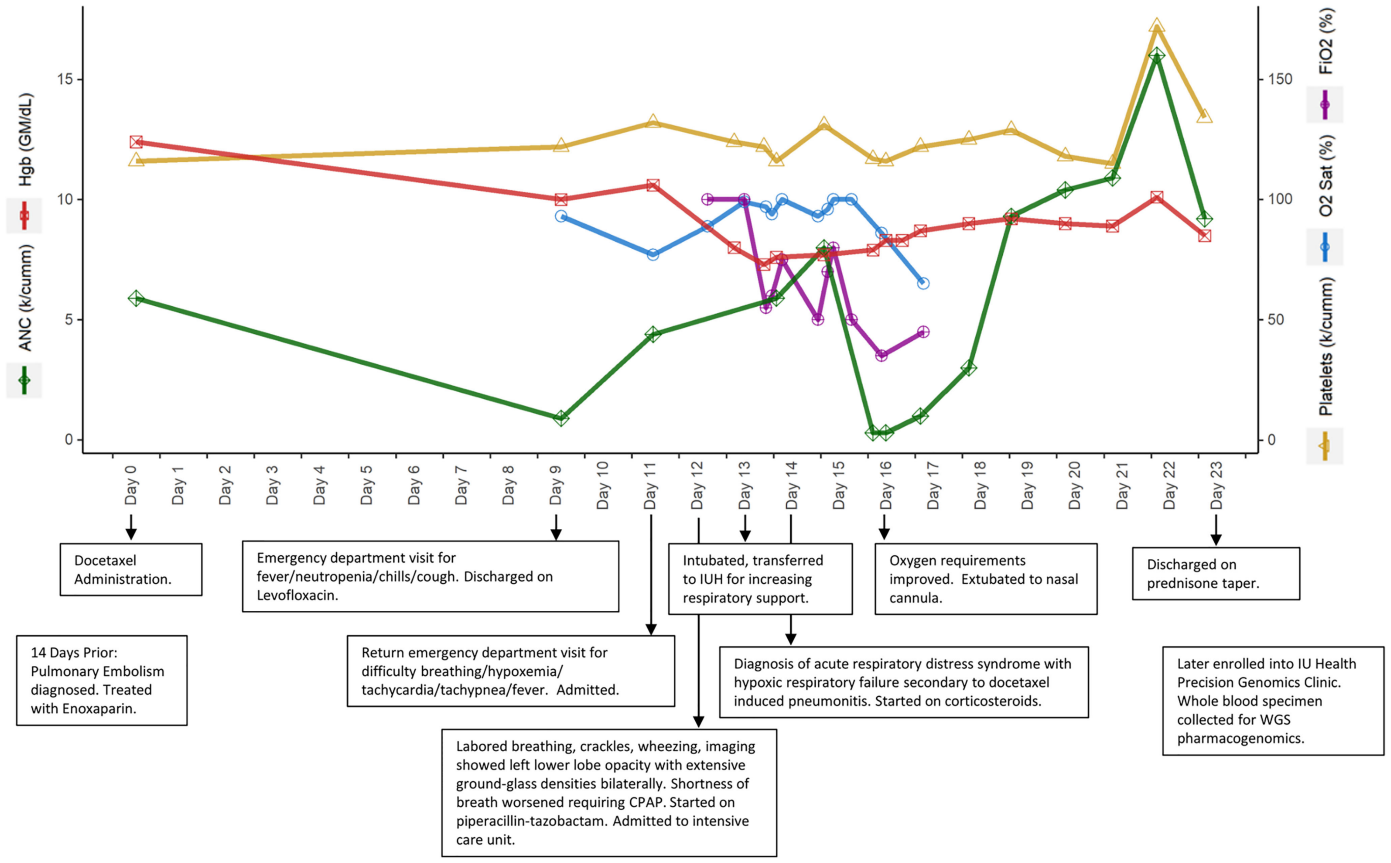
Timeline of events and select lab values for patient 1.

Patient 1 is a 52-year-old male with metastatic prostate cancer who was administered a single dose of docetaxel 75 mg/m^2^. Nine days later, he presented to the emergency department with complaints of fever, chills, hemoptysis, and was grade 3 febrile neutropenic. He was discharged on levofloxacin for suspected community-acquired pneumonia. On day 11, he returned to the emergency department and was admitted for acute respiratory distress accompanied by fever, hypoxemia, tachypnea, and tachycardia. On day 12, the respiratory distress worsened and he was moved to the intensive care unit. On day 13, he was intubated due to hypoxic respiratory failure secondary to docetaxel-induced pneumonitis and was started on corticosteroids. Neutropenia progressed to grade 4 by day 16. By day 23, the patient was weaned off respiratory support and discharged. The timeline of events is shown in **Figure 1**. Noteworthy past medical history includes diagnosis of a pulmonary embolism 14 days prior to docetaxel treatment, which was treated with enoxaparin and considered resolved. Aspartate aminotransferase (AST), alanine aminotransferase (ALT), and total bilirubin were normal throughout the time course. Alkaline Phosphatase was elevated to 162 U/L on day 13. These labs indicate that general liver function was not impaired or diseased at the time of docetaxel administration. The patient was not concomitantly prescribed any CYP3A inhibitors.

The pneumonitis, dyspnea, and neutropenia were grade 4 toxicities caused by one docetaxel dose. Although the patient’s earlier pulmonary embolism may have been a predisposing factor to the pneumonitis, not all patients with a history of pulmonary emboli who receive docetaxel develop life-threatening toxicity; therefore, we investigated potential underlying genetic defects in docetaxel clearance.

Our *CYP3A* genetic analyses (see Appendix for methods and **Table 1** for results) of this patient revealed the following: (1) homozygous CYP3A4*22, which causes reduced expression of the active enzyme (26) and is associated with increased docetaxel-related adverse events (9, 10); (2) heterozygous CYP3A4*3, a rare reduced-function missense variant (22–24); and (3) homozygous CYP3A5*3, a common loss of function splicing defect that has been associated with increased docetaxel exposure and adverse events (9, 10). All genotyping and sequencing was carried out in a phenotype-blind process.

**Table 1:** Patient status for genetic variants in docetaxel pharmacokinetics-related genes that have previously been associated with exposure or toxicity.

Gene	Variant	Patient 1	Patient 2	Variant Allele Frequency^+^	Effect on Docetaxel Exposure or Toxicity:	
					Exposure (Reference)	Toxicity (Reference)
ABCB1	rs1045642	HT	HM	0.49	↓ (11)	↓ (12, 13)
ABCB1	rs1128503	HT	HM	0.57	↓ (14)	↔ (15)
ABCB1	rs2032582	HT	HM	0.56	↓ (13)	↑ (15)
ABCC1	rs246221	HT	HT	0.31	NS	↑ (16)
ABCC2	rs12762549	HT	HT	0.45	↔^†^ (17)	↑ (18)
ABCC10	rs2125739	HT	HT	0.25	NS	↓ (19)
CAR	rs2501873	WT	HM	0.44	↔ (20)	↑ (20)
CAR	rs2502815	WT	HM	0.26	↔ (20)	↓ (20)
CYP3A4	rs2740574	HM	HM	0.89	↔ (6)	↓ (12, 21)
CYP3A4	*3 (rs4986910)	HT	WT	<0.01	↑ *in vitro* (22–24)	NS
CYP3A4	*22 (rs35599367)	HM	HM	0.04	NS	↑ (9, 10)
CYP3A5	*3 (rs776746)	HM	HM	0.89	↑ (6)	↑ (9, 10)
HNF4A	rs1800963	HT	HT	0.52	↓ (20)	↔ (20)
HNF4A	rs2273618	HT	HM	0.58	↓ (20)	↑ (20)
HNF4A	rs3746574	HT	HM	0.50	↓ (20)	↑ (20)
HNF4A	rs6103731	HT	HM	0.57	↓ (20)	↔ (20)
PXR	rs3732359	HM	HM	0.74	↔ (20)	↑ (20)
PXR	rs3732360	HM	HM	0.71	↔ (20)	↑ (20)
RXRA	rs3132291	HM	HT	0.67	↑ (20)	↔ (20)
RXRA	rs4240711	HT	HT	0.58	↓ (20)	↔ (20)
RXRA	rs4842198	HM	WT	0.71	↑ (20)	↔ (20)
RXRA	rs7861779	WT	HM	0.23	↑ (20)	↔ (20)
SLCO1B3	rs3834935	WT	HT	0.14	↑ (25)	NS
SLCO1B3	rs4149117	HM	HM	0.84	↔ (25)	NS
SLCO1B3	rs4149118	HM	HT	0.65	↔ (25)	NS
SLCO1B3	rs7311358	HM	HM	0.83	↑ (25)	↔ (18)
SLCO1B3	rs11045585	WT	HM	0.13	↑ (25)	↑ (18)

Key: ↑, findings of increased exposure or toxicity; ↓, findings of decreased exposure or toxicity; ↔, findings of no change in exposure or toxicity; NS, not studied; HM, homozygous variant; HT, heterozygous; WT, homozygous wild-type.

Genotypes in blue are predicted to reduce docetaxel-related adverse events when considering pattern of inheritance.

Genotypes in red are predicted to increase docetaxel-related adverse events when considering pattern of inheritance.

Genotypes in black are predicted to not effect docetaxel-related adverse events when considering pattern of inheritance.

Variants shaded in the same color are contained within haplotypes, as defined below.

**Haplotype Definitions (defined by D’≥0.99)**:

1.) CYP3A4 & CYP3A5: rs35599367 (CYP3A4*22) variant allele (A) predicts rs776746 (CYP3A5*3) variant allele (C) (D’=1.0) and rs2740574 variant allele (A) (D’=1.0).

2.) HNF4A: rs6103731 variant allele (G) predicts rs2273618 variant allele (C) (D’=0.99).

3.) PXR: rs3732360 variant allele (T) predicts rs3732359 variant allele (A) (D’=1.0)

4.) SLCO1B3: rs4149118 variant allele (A) predicts rs7311358 variant allele (A) (D’=1.0), rs4149117 variant allele (G) (D’=1.0), and rs11045585 wild-type allele (A) (D’=0.99).

**Footnote:**

^+^Variant allele frequencies are based on total global population frequencies from the Allele Frequency Aggregator (ALFA) project and as displayed on the Single Nucleotide Polymorphism Database (dbSNP).

†A trend was reported towards ABCC2 rs12762549 being associated with reduced docetaxel clearance (observed p-value was 0.048, but the multiple comparison-adjusted significance threshold was p=0.025.

Since CYP3A4*22 is a rare functional variant that was a plausible cause of the docetaxel toxicity, we searched our research cohort for additional CYP3A4*22 homozygotes that received docetaxel. We identified only one additional patient who was homozygous for CYP3A4*22. She was also homozygous for CYP3A5*3, but did not have the CYP3A4*3 variant. She was a 64-year old female breast cancer patient who was treated with docetaxel and cyclophosphamide for a total of 4 cycles. She was also prophylactically treated with granulocyte colony stimulating factor drugs with each cycle and darbepoetin alfa after her 1^st^ and 4^th^ cycle. Over the course of 4 cycles she experienced grade 3 leukopenia, grade 2 anemia, generalized weakness, erythema on her extremities, and numbness in her extremities indicating peripheral neuropathy. We believe her toxicity could be regarded as severe and was caused by docetaxel.

Given the genomic proximity of *CYP3A4* and *CYP3A5* and the existence of known haplotypes containing functional variants in both genes (27), we conducted linkage disequilibrium (LD) analyses on the these *CYP3A* variants. Using the global population data from the 1000 Genomes Project, we used LDpair (28) to determine the linkage disequilibrium between CYP3A4*22, CYP3A4*3, and CYP3A5*3. CYP3A4*22 always occurred with CYP3A5*3 (**Supplemental Table S1**). Also, 62% of the CYP3A4*3 alleles occurred with CYP3A4*22 (**Supplemental Table S2**). Thus, this is a rare haplotype containing reduced-function *CYP3A* variants that appears to be associated with docetaxel exposure and toxicity.

In our analysis of other pharmacokinetic genes, both patients were heterozygous for *ABCC2* rs12762549, which has been associated with docetaxel-related leukopenia (18). Both patients had variants in *HNF4A*, *PXR*, and *RXRA* that have been weakly associated with increased exposure and/or toxicity, and patient 2 was homozygous for *SLCO1B3* rs11045585. All pharmacokinetic variants potentially involved in these cases are reviewed in **Table 1**. Analysis of pharmacodynamic-related variants using Qiagen Clinical Insights did not identify “pathogenic” or “likely pathogenic” variants associated with observed docetaxel toxicities.

## Discussion

The observations presented in this case report implicate the reduced-function variants in *CYP3A* in the severe docetaxel-induced toxicities. First, CYP3A4 is the main route of docetaxel detoxification and elimination; second, the CYP3A4*22 variant reduces CYP3A4-mediated drug clearance; third, it is a rare variant (0.2% of people are homozygous for CYP3A4*22) (29); fourth, the only two patients in our research cohort that were CYP3A4*22 homozygotes and received docetaxel developed severe toxicity; and fifth, the patient that also had the rare CYP3A4*3 variant (variant allele frequency < 0.01) had even greater toxicities. Both patients were also homozygous for the CYP3A5*3 variant, which causes loss of CYP3A5 function. Previous studies have linked increased docetaxel exposure to toxicity, which provides mechanistic plausibility. Since docetaxel is cleared by the time these toxicities occur, and it would not be appropriate to rechallenge the patient with docetaxel, it is not feasible to obtain plasma docetaxel concentrations after the toxicities have occurred; this is a limitation of this observation. Also, we found no concurrent medications that inhibit CYP3A activity, suggesting it may be a genetic cause and we did not find any variants linked to pharmacodynamic sensitivity to docetaxel toxicities.

We searched the literature for reports of additional CYP3A4*22 homozygous patients who received docetaxel, and found only one report by Sim et al.; that patient died from typhlitis induced by a single dose of docetaxel (9). Thus, we are aware of a total of 3 cases of CYP3A4*22 homozygotes that were treated with docetaxel; all three experienced severe toxicities, one of which was fatal and one of which was life-threatening.

Several studies have reported the association of heterozygous CYP3A4*22 carriers with docetaxel-induced toxicities. Gastric cancer patients who were CYP3A4*22 carriers had higher rates of toxicity (58%) compared to non-carriers who were homozygous for CYP3A5*3 (41%) and non-carriers heterozygous for CYP3A5*3 (17%) (10). Increased docetaxel-induced adverse events were also observed in breast cancer patients who were CYP3A4*22 carriers and CYP3A5*3 homozygotes (9). Most of those toxicities were less severe than those we report in the current study, as would be expected in carriers of only one loss of function CYP3A4*22 allele. A role of CYP3A metabolism in docetaxel toxicities is also supported by the FDA label recommendations to avoid the concomitant use of strong CYP3A4 inhibitors (1).

Additional factors may also contribute to the toxicities. For example, the two patients in this report also had genetic variants in *SLCO1B3* and *ABCC2* transporters, which have been associated with docetaxel toxicities (18). They also had genetic variants in additional *CYP* regulatory genes that have been weakly associated with docetaxel toxicities (20). Patient 2 was homozygous for variants in *ABCB1* that have been associated with reduced exposure, potentially compensating for the loss in function of CYP3A (11–15). The previous pulmonary embolism in patient 1 may also have contributed to the pneumonitis. Considered together, the final toxicity phenotypes are likely composed of the net effect of all these genetic and environmental factors.

Taxane-induced pneumonitis has been speculated to occur through an immune-mediated delayed hypersensitivity reaction. Although patient 1’s pneumonitis could have been allergic in nature, evidence also supports the possibility of it being caused by increased docetaxel exposure. Docetaxel-induced interstitial pneumonitis is estimated to occur in about 1 to 5% of patients and is known to be dose-dependent, occurring more frequently among those treated with 100mg/m^2^ vs. 60mg/m^2^ (2.2% vs. 0.7%) (30); but, allergic reactions are not typically dose-dependent. The patient had grade 4 neutropenia, a symptom frequently observed in patients with genetic variants resulting in increased docetaxel exposure (10), but not typically observed in docetaxel hypersensitivity reactions (31). Also, immune-mediated hypersensitivity often requires sensitization periods; however, in patient 1 and in some other case reports, the toxicity occurs after a single dose (32, 33). It is possible that the reduced clearance may sustain single-dose docetaxel exposure long enough to allow for allergic sensitization to occur. There may also be genetic variability in immune function genes that result in increased susceptibility to docetaxel toxicities, although those have yet to be identified.

In conclusion, our case report adds additional evidence to support the role of pharmacokinetic genetic variants in docetaxel toxicity. Since we are not aware of any cases where docetaxel was safely given to cancer patients with two reduced-function *CYP3A4* alleles, we propose that extreme caution should be used when treating those patients with docetaxel.

## Author's Note

This report contains original work that was presented as a poster at the Pharmacogenomics Global Research Network – American Society of Human Genetics (PGRN-ASHG) Conference, October, 2021.

## Data Availability Statement

The original contributions presented in the study are included in the article/**Supplementary Material**. Further inquiries can be directed to the corresponding author.

## Ethics Statement

The studies involving human participants were reviewed and approved by Indiana University Institutional Review Board. The patients/participants provided their written informed consent to participate in this study. Written informed consent was obtained from the individual(s) for the publication of any potentially identifiable images or data included in this article.

## Author Contributions

NP, TS, RL, and TCS wrote manuscript and performed clinical/genetic investigation. CA, MR, and BS performed clinical/genetic investigation. All authors contributed to the article and approved the submitted version.

## Funding

This study was supported with funding from NIH Grant R35GM131812 (TCS), T32GM008425 (NP), the Monogrammed Medicine Program of the Vera Bradley Center for Breast Cancer Research (TCS), and the Indiana University Grand Challenge Precision Health Initiative (TCS).

## Conflict of Interest

The authors declare that the research was conducted in the absence of any commercial or financial relationships that could be construed as a potential conflict of interest.

## Publisher’s Note

All claims expressed in this article are solely those of the authors and do not necessarily represent those of their affiliated organizations, or those of the publisher, the editors and the reviewers. Any product that may be evaluated in this article, or claim that may be made by its manufacturer, is not guaranteed or endorsed by the publisher.
