# Supplementary material for: Life-Threatening Docetaxel Toxicity in a Patient With Reduced-Function CYP3A Variants: A Case Report
Source: Front Oncol. 2022 Jan 31;11:809527. doi: 10.3389/fonc.2021.809527 (PMC8841796; doi:10.3389/fonc.2021.809527)
Supplement: Supplementary file 2 [file DataSheet_2.docx]

**Appendix**

**Study Design**

This study was approved by the Indiana University Institutional Review Board. Informed consent was obtained to have patient-derived specimens and medical records used for research and reporting. Patients’ germline DNA was obtained from whole blood and used for whole-genome sequencing (WGS), whole-exome sequencing (WES), targeted pharmacogenetics (PGx) genotyping, and Sanger sequencing.

Integrated Genomics Viewer Version 2.8.13 (Broad Institute, Cambridge, MA)^1^ was used to visualize WGS data, and Qiagen Clinical Insight Interpret Translational (Qiagen, Germantown, MD) was used to screen for “pathogenic” or “likely pathogenic” pharmacodynamic-related variants associated with docetaxel adverse events using the following biological filters: “grade 3-4 leukopenia,” “grade 4 leukopenia,” “interstitial pneumonitis,” “leukopenia,” “life-threatening interstitial pneumonitis,” “neutropenia,” “neutropenia acquired type,” and “serious interstitial pneumonitis.” Aldy v3.2 was used for PGx genotyping from WGS^2^. Additional variants within docetaxel pharmacokinetic genes, as determined by PharmGKB (https://www.pharmgkb.org/chemical/PA449383/clinicalAnnotation), were pulled from the VCF files using bedtools (v2.30.0) intersect (https://bedtools.readthedocs.io/en/latest/content/tools/intersect.html).

**Clinical Whole-Genome and Exome Sequencing**

For patient 1 and 2, germline and somatic WGS and WES was performed by NantOmics (Culver City, CA), a CLIA-certified laboratory, as previously described^3^ for clinical genome-guided therapy at the Indiana University Precision Genomics Clinic (Indianapolis, IN). Sequencing depth across the *CYP3A4* and *CYP3A5* genes was ≥ 30× in both germline and somatic WGS and > 100x across all exons of *CYP3A4* and *CYP3A5* genes in both germline and somatic WES. *CYP3A4*22* (rs35599367) intronic position was > 30x coverage in the germline WES.

**Whole-Genome Sequencing**

For patient 2, the subject’s DNA was obtained and used for WGS performed by the Center for Medical Genomics at Indiana University (Indianapolis, IN). Illumina DNA PCR-Free Library Prep Kit was used to prepare the WGS libraries according to the manufacturer’s instructions. In short, 300ng gDNA was tagmented using the bead-linked transposomes, followed by post tagmentation cleanup and sample index ligation. The resulting libraries were purified using a double-sided bead purification procedure. Qubit ssDNA assay kit (Thermo Fisher Scientific, Waltham, MA) was used to quantify the final single-stranded libraries. Prepared libraries were then sequenced on Illumina NovaSeq 6000 using S4 flow cell with a paired-end read length of 2 × 150 bp and dual 10 bp index reads. Paired-end sequence reads were first processed to remove Illumina adapter sequences and low-quality base calls using Trim Galore (http://www.bioinformatics.babraham.ac.uk/projects/trim_galore/). High-quality reads were aligned to human reference genome hg19 using BWA-MEM (v0.7.15).^4,5^ Sentieon version 201911.01 (Sentieon, Inc, https://www.sentieon.com/) was used for variant detection.^6^ Sequencing depth across the *CYP3A4* and *CYP3A5* genes was ≥ 30× in WGS.

**Targeted PGx genotyping and Sanger Sequencing**

For patient 1, targeted PGx genotyping for *CYP3A4* and *CYP3A5* as well as Sanger sequencing was performed by the CAP-accredited, CLIA-certified IU Pharmacogenomics Laboratory (Indianapolis, IN). DNA was extracted from whole blood using the Qiagen EZ1 (Germantown, MD) according to manufacturer's protocol. DNA samples were analyzed using the QuantStudio 12K Flex (software v1.2.2; Waltham MA) and subjected to Taqman® allele discrimination using either individual reagents or in a custom-designed OpenArray® format (Thermo Fisher Scientific, Waltham, MA). Genomic DNA was amplified and mixed with dual-labeled oligonucleotides that hybridize to a specific target sequence. Hydrolysis by the 5’-3’ exonuclease activity of Taq polymerase releases the fluorescent reporter signal, permitting quantitative measurement of the accumulation of the PCR product via the fluorophore signal. Software utilized includes Genotyper (v1.3) (Thermo Fisher Scientific, Waltham, MA) and Alleletyper™ (1.0) (Thermo Fisher Scientific, Waltham, MA). DNA was also Sanger sequenced for the *CYP3A4*3* variant (rs4986910) using BigDye™ Terminator v3.1 (Thermo Fisher Scientific, Waltham, MA) and run on Applied Biosystems, Inc. 3500xL (Thermo Fisher Scientific, Waltham, MA). Software utilized was Mutation Surveyor V4.0.7 (SoftGenetics, State College, PA). The sequences of the primers used are described in the table below, in addition to the Sanger sequencing result that confirms the *CYP3A4*3* heterozygous variant identified from the patient’s germline WGS (Appendix Figure 1).

**Appendix Table 1**: Primer sequences used for Sanger Sequencing of *CYP3A4*3* (dbSNP rs4986910)

| Primer Name | Primer sequence with M13 Tails for Sanger (5’ to 3’) |
| --- | --- |
| CYP3A4*3 Forward 1 | 5’-TGTAAAACGACGGCCAGTGGACACATCACCACCCTGAA-3’ |
| CYP3A4*3 Reverse 1 | 5’-CAGGAAACAGCTATGACCTCTTTGGCCCAGAGAACAAA-3’ |
| CYP3A4*3 Forward 2 | 5’-TGTAAAACGACGGCCAGTAGTGTGACTTGAGGCAGGAA-3’ |
| CYP3A4*3 Reverse 2 | 5’-CAGGAAACAGCTATGACCTCCAGAACTGAAGCACCCTT-3’ |

*Underlined nucleotides indicate the M13 tail*


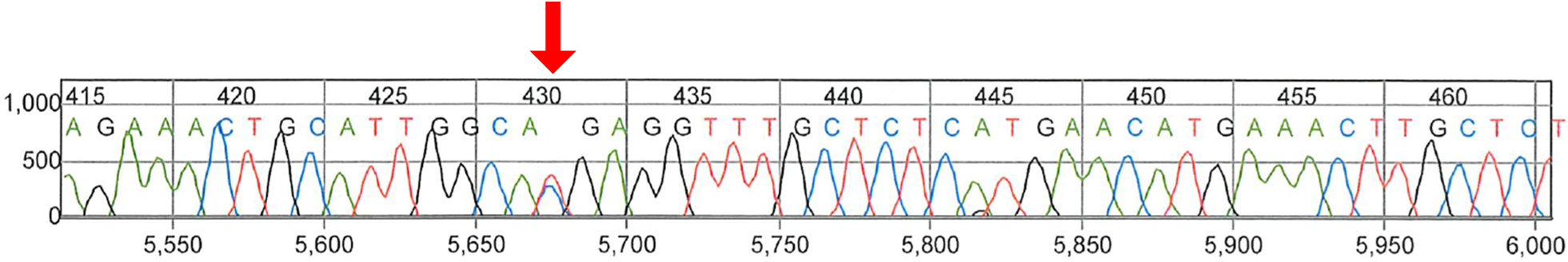


**Appendix Figure 1**: Sanger sequencing confirmed the presence of the heterozygous *CYP3A4*3* (c.1334T>C, p.M445T) variant in the germline genome (T>C on the − coding strand) as identified by the arrow.

**ALDY Pharmacogenomics Genotyping**

Aldy v3.2 was operationalized in LifeOmic’s Precision Health Cloud™ and Indiana University’s research supercomputer, Carbonate, to genotype pharmacogenomic star alleles and diplotypes from WGS using the BAM file as input to genotype for *CYP3A4* and *CYP3A5* for both patients.

**Appendix References**

1. Robinson JT, Thorvaldsdottir H, Winckler W, et al. Integrative genomics viewer. Nat Biotechnol 2011;29:24-6.

2. Numanagic I, Malikic S, Ford M, et al. Allelic decomposition and exact genotyping of highly polymorphic and structurally variant genes. Nat Commun 2018;9:828.

3. Rabizadeh S, Garner C, Sanborn JZ, Benz SC, Reddy S, Soon-Shiong P. Comprehensive genomic transcriptomic tumor-normal gene panel analysis for enhanced precision in patients with lung cancer. Oncotarget 2018;9:19223-32.

4. Li H, Durbin R. Fast and accurate short read alignment with Burrows-Wheeler transform. Bioinformatics 2009;25:1754-60.

5. Li H. Aligning sequence reads, clone sequences and assembly contigs with BWA-MEM. 2013.

6. Kendig KI, Baheti S, Bockol MA, et al. Sentieon DNASeq Variant Calling Workflow Demonstrates Strong Computational Performance and Accuracy. Front Genet 2019;10:736-.
