# Supplementary material for: Life-Threatening Docetaxel Toxicity in a Patient With Reduced-Function CYP3A Variants: A Case Report
Source: Front Oncol. 2022 Jan 31;11:809527. doi: 10.3389/fonc.2021.809527 (PMC8841796; doi:10.3389/fonc.2021.809527)
Supplement: Supplementary file 3 [file Image_1.pdf]

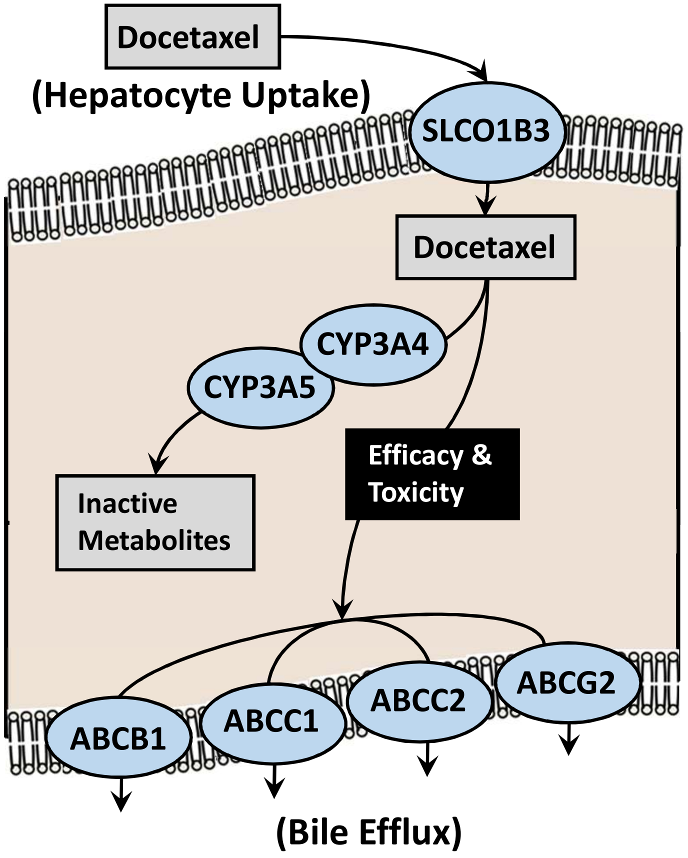

Supplemental Figure S1: Clearance pathway for docetaxel.
This figure was adapted from its original form under the PharmGKB Creative Commons Attribution-ShareAlike 4.0 International License.
