# Supplementary material for: Life-Threatening Docetaxel Toxicity in a Patient With Reduced-Function CYP3A Variants: A Case Report
Source: Front Oncol. 2022 Jan 31;11:809527. doi: 10.3389/fonc.2021.809527 (PMC8841796; doi:10.3389/fonc.2021.809527)
Supplement: Supplementary file 4 [file Table_1.docx]

Supplemental Table S1: LD Pair results showing global population allele counts for the two variants. CYP3A4*22 was only present if CYP3A5*3 was also present, indicating that CYP3A4*22 may exist only on the CYP3A5*3 allele.

| ***CYP3A5*3* vs.**  ***CYP3A4*22*** | | **rs35599367**  **(*CYP3A4*22*)** | |  |
| --- | --- | --- | --- | --- |
|  |  | **G** | **A**  **(variant)** | **Total** |
| **rs776746**  **(*CYP3A5*3*)** | **T** | 1896 | 0 | 1896 |
|  | **C**  **(variant)** | 3037 | 75 | 3112 |
|  | **Total** | 4933 | 75 | N=5008 alleles |

Supplemental Table S2: LD Pair results showing global population allele counts for the two variants. CYP3A4*3 was present in 11% (8/75) of alleles with CYP3A4*22, but only 0.1% (5/4933) of alleles without CYP3A4*22, indicating the existence of a haplotype with both CYP3A4*3 and CYP3A4*22.

| ***CYP3A4*3* vs.**  ***CYP3A4*22*** | | **rs35599367**  **(CYP3A4*22)** | |  |
| --- | --- | --- | --- | --- |
|  |  | **G** | **A**  **(variant)** | **Total** |
| **rs4986910**  **(CYP3A4*3)** | **A** | 4928 | 67 | 4995 |
|  | **G (variant)** | 5 | 8 | 13 |
|  | **Total** | 4933 | 75 | N=5008 alleles |
